# Olfactory Dysfunction following COVID-19 and the Potential Benefits of Olfactory Training

**DOI:** 10.3390/jcm12144761

**Published:** 2023-07-18

**Authors:** Abdullah A. Alarfaj, Abdulrahman Khalid Aldrweesh, Alghaydaa Fouad Aldoughan, Sumaia Mohammed Alarfaj, Fatimah Khalid Alabdulqader, Khalid A. Alyahya

**Affiliations:** 1 Otolaryngology Unit, Department of Surgery, King Faisal University, Al-Ahsa 31982, Saudi Arabia; kalyahya@kfu.edu.sa; 2 College of Medicine, King Faisal University, Al-Ahsa 31982, Saudi Arabia; dr.aldrweesh@gmail.com (A.K.A.); sumaiaalarfaj99@gmail.com (S.M.A.);

**Keywords:** COVID-19, vaccine, olfactory dysfunction, chemosensory dysfunction, anosmia, hyposmia, parosmia, olfactory exercise, adherence

## Abstract

COVID-19 is associated with a common symptom of olfactory dysfunction, which may persist even after the infection is resolved. Olfactory training (OT) has emerged as the most effective intervention for post-viral olfactory dysfunction. OT involves daily exposure of the olfactory system to various odors. The current study aims to explore olfactory dysfunction following COVID-19 and the potential benefits of olfactory training. Methods: This is a cross-sectional study conducted among adults aged 18–60 living in Alahssa, Saudi Arabia. An online questionnaire containing an informed consent form and a survey to collect demographic data, vaccination status, level of loss of smell and taste, and the level of awareness about olfactory training (OT) was distributed among all participants who agreed to participate in this study. Results: The study included 524 participants and presented their baseline characteristics, including age, gender, COVID-19 infection status, and complaints. Most patients were female (66.0%), and 46.2% had previously been infected with COVID-19. About 54.8% of participants reported chemosensory dysfunction, while 286 had olfactory dysfunction. Of those, 29.8% had anosmia, 16.8% had hyposmia, and 8.0% had parosmia. Results suggest that being fully or partially vaccinated may offer some protection against olfactory dysfunction compared to being unvaccinated. Adherence to olfactory training was associated with improvement in the sense of smell. Conclusions: The study highlights the importance of awareness and adherence to olfactory training, which may improve the sense of smell in individuals with chemosensory dysfunction. The findings of this study can inform public health policies and interventions aimed at reducing the burden of olfactory dysfunction associated with COVID-19 vaccination.

## 1. Introduction

The COVID-19 pandemic has caused a global health crisis, affecting millions of people worldwide and leading to a wide range of symptoms and complications [1,2,3]. One of the most common symptoms reported by COVID-19 patients is a loss of sense of smell, also known as anosmia [4]. Anosmia can have a significant impact on a patient’s quality of life, as it can affect their ability to detect dangerous odors, enjoy food, and even recognize loved ones [5,6]. Although the exact mechanisms behind anosmia following COVID-19 are not yet fully understood, recent studies have suggested that the virus may directly damage the olfactory neurons in the nose, affecting the sense of smell [7].

The sense of smell, or olfaction, is a complex process that involves the detection of odors by olfactory receptors located in the nasal cavity [8]. These receptors are specialized cells that respond to specific odor molecules and send signals to the olfactory bulb, a neural structure located at the front of the brain [9]. The olfactory bulb then sends signals to various brain regions that are involved in processing smell, including the amygdala, hippocampus, and orbitofrontal cortex. These regions are responsible for identifying the quality, intensity, and location of the odor [10,11]

When the olfactory neurons in the nose are damaged or destroyed, the sense of smell can be impaired or lost. This can be caused by a variety of factors, including viral infections, head injuries, and exposure to certain chemicals or drugs [12]. The virus is known to enter cells by binding to the ACE2 receptor, which is found in various tissues in the body, including the olfactory epithelium in the nose [13,14]. Once inside the cells, the virus may cause damage to the olfactory neurons, leading to inflammation and cell death [15].

In addition to direct damage to the olfactory neurons, COVID-19 may also affect the olfactory system indirectly through systemic inflammation and immune activation [16]. COVID-19 is known to cause a robust immune response, leading to the release of cytokines and other inflammatory molecules [17]. These molecules can cross the blood–brain barrier and affect the olfactory system, leading to inflammation and damage to the olfactory neurons [18]. The olfactory system is unique in that it has the ability to regenerate olfactory neurons throughout life [19]. However, the extent to which olfactory neurons can regenerate following damage is still not fully understood. Some studies have suggested that olfactory neurons can regenerate in response to injury, while others have shown that the ability to regenerate declines with age [20,21]

In the olfactory system, glial cells, including microglia, play a crucial role in supporting and protecting the neurons involved in the sense of smell. These specialized immune cells are responsible for maintaining the overall health and functioning of the olfactory system. Emerging evidence suggests that glial cells, particularly microglia, may be involved in the response to COVID-19 infection in the olfactory system [22,23]. It is believed that these cells may become activated or potentially damaged as a result of the viral invasion. Such activation or damage to glial cells could contribute to the dysfunction of olfactory neurons, leading to the loss of sense of smell observed in COVID-19 patients [24].

Understanding the mechanisms behind olfactory dysfunction following COVID-19 is crucial for developing effective treatments for affected patients [25]. In addition to Olfactory training, which has shown promising results in improving olfactory function, other treatment options may include medications, surgery, or other interventions, depending on the underlying cause of the problem [26]. More research is needed to determine the effectiveness of these treatments and to identify new approaches for treating olfactory dysfunction following COVID-19 [27]. One promising approach to treating olfactory dysfunction following COVID-19 is olfactory training [28]. Olfactory training involves repeated exposure to different smells to help stimulate the olfactory neurons and improve olfactory function [29]. The goal of olfactory training is to help the brain “relearn” how to process smells and improve the sense of smell over time [30].

In a study published in the European Archives of Oto-Rhino-Laryngology, olfactory training was found to be effective in improving olfactory function in patients with post-viral anosmia, with significant improvements observed after 12 weeks of training [31]. Olfactory training is a simple and low-cost intervention that can be done at home, making it an attractive option for patients with olfactory dysfunction following COVID-19 [32]. However, it is important to note that olfactory training may not be effective for all patients, and more research is needed to determine its efficacy in COVID-19-related anosmia [32].

The prevalence of olfactory dysfunction following COVID-19 highlights the need for healthcare providers to be aware of this symptom and to provide appropriate care to affected patients. In addition to olfactory training, other treatment options for olfactory dysfunction may include medications, surgery, or other interventions, depending on the underlying cause of the problem. Public health measures such as mask-wearing and social distancing may also help reduce the spread of COVID-19 and prevent olfactory dysfunction in the first place. However, it is important to note that even with these measures in place.

Olfactory training has been found to have a significant impact on the recovery of olfactory function in patients suffering from post-viral olfactory dysfunction, including those with COVID-19 [32]. This training involves the regular exposure to a set of odorants in a structured way, with the aim of stimulating the regeneration of olfactory receptor neurons and the formation of new synapses in the olfactory bulb [33].

The underlying neurological mechanism of olfactory training has been extensively investigated, and it is thought to involve the process of neuroplasticity, which is the ability of the brain to change its structure and function in response to sensory input and experience [34]. Neuroplasticity is a key feature of the olfactory system, which has a remarkable capacity for regeneration and repair [35].

In addition to the promotion of neuroplasticity, olfactory training has been found to increase the connectivity between the olfactory bulb and other brain regions involved in olfactory perception, such as the piriform cortex and the orbitofrontal cortex [36]. This increased connectivity may help to improve the processing of odorants and enhance the perception of smell.

Furthermore, olfactory training has been found to have an impact on the emotional processing of odorants, which is mediated by the amygdala and other limbic regions of the brain [37]. Patients with post-viral olfactory dysfunction may experience emotional distress as a result of their loss of smell, and olfactory training has been found to help alleviate some of this distress by promoting the positive association between odorants and emotional states [38].

The aim of this study was to investigate the neurobiology of olfactory dysfunction following COVID-19 and explore the potential benefits of olfactory training. Specifically, we aimed to estimate the prevalence of COVID-19-related olfactory dysfunctions, including anosmia, hyposmia, and parosmia, among patients in AlAhsa, Saudi Arabia. Additionally, we sought to assess the level of awareness and adherence to olfactory training among the study participants. Understanding the olfactory dysfunction following COVID-19 is crucial for several reasons. It provides insights into the pathophysiological mechanisms underlying this symptom, helping to unravel the complex interplay between the virus and the olfactory system. It sheds light on the potential long-term consequences of COVID-19 on the olfactory system and the need for appropriate management strategies. Lastly, investigating the potential benefits of olfactory training, such as olfactory training, can contribute to the development of effective interventions to improve the sense of smell in individuals with olfactory dysfunction.

## 2. Materials and Methods

### 2.1. Study Design

A cross-sectional study design was used to investigate olfactory dysfunction following COVID-19 and the potential benefits of olfactory training in Al Ahsa, Saudi Arabia. The study aimed to estimate the prevalence of COVID-19-related anosmia, hyposmia, and parosmia among patients in Alahssa City and establish the level of awareness of smell training. Participants were recruited through convenience sampling from healthcare centers and hospitals in Al Ahsa. The study sample was representative of the population in Al Ahsa, Saudi Arabia, based on age and gender distributions.

### 2.2. Data Collection

Data were collected from 10 January 2022 to 16 February 2022, using a self-reported retrospective study design. A standardized questionnaire was used to collect data, which was distributed through a “Google Form” and paper-based questionnaires. The questionnaire included demographic information, COVID-19 vaccination status, level of loss of smell and taste, and the level of awareness about smell training. Multiple methods for data collection were used to ensure accessibility to all potential participants.

### 2.3. Sample Size

The sample size for this study was determined to be 535 COVID-19-infected patients. The sample size calculation was based on a 95% confidence interval and a margin of error of 5%, with consideration for a potential non-response rate. Participants were included in the study if they were infected with COVID-19 that was confirmed via PCR test and recorded on their personal health record in Sehhaty, a “Platform which is the unified platform of Ministry of Health, which allows users to access health information and obtain several health services provided by various entities in the health sector in Saudi Arabia”, were aged between 18 and 60 years, and fully answered the questionnaires. Those who did not meet the inclusion criteria or who had missing data were excluded from the study.

### 2.4. Data Analysis

Data analysis in this study involved the use of descriptive statistics, such as frequency and percentage distributions, to summarize the prevalence of COVID-19-related anosmia, hyposmia, and parosmia among patients in Alahssa City. Multivariable logistic regression was used to adjust for potential confounding variables, such as age, gender, and COVID-19 vaccination status. Chi-square tests were also used to determine the association between variables. The results of the analysis were presented using tables and graphs, with appropriate measures of central tendency and variability reported. The findings were interpreted with caution, taking into account the limitations of the study and potential sources of bias.

### 2.5. Procedure

The olfactory training was explained to participants who reported olfactory dysfunction through a leaflet that was provided to them. The leaflet contained detailed instructions on how to perform the training at home. Participants were instructed to smell different scents, such as essential oils or spices, and try to identify them. The scents were presented in a random order to prevent memorization.

Participants were advised to perform the training for a minimum of 10 min each day, preferably in the morning or early afternoon, to increase the chances of success. They were encouraged to repeat the training daily for at least 3 months to achieve optimal results. A variety of odors were used during the olfactory training. Participants were provided with a range of scents, such as essential oils or spices, to smell and identify as part of their training regimen. The specific odors used in the study varied and were tailored to the individual preferences and availability of the participants.

Follow-up was conducted after two weeks of undergoing olfactory testing. Participants were contacted either through phone or email to determine if they had noticed any improvements in their sense of smell. They were asked to rate their level of improvement on a scale of 0 to 10, with 0 indicating no improvement and 10 indicating complete improvement.

Participants who did not report any improvement after two weeks of olfactory testing were advised to continue the testing for an additional two weeks and were followed up again. If participants did not experience any improvement after 12 weeks of olfactory testing [32,39], they were referred to an otolaryngologist for further evaluation and management.

It was emphasized to all participants that the olfactory training was intended to supplement medical treatment, not replace it. They were advised to continue any medications prescribed by their healthcare provider and to seek medical attention if their symptoms worsened or did not improve.

## 3. Results

Table 1 presents the baseline characteristics of the patients included in the study, which had a sample size of 524. The table provides a breakdown of the frequencies and percentages of different variables such as age, gender, COVID-19 infection status, and complaints reported by the patients. The age distribution of the participants shows that 40.3% of patients were in the age range of 18–25 years, followed by 23.3% in the age range of 36–45 years. Most of the participants were female, with 66.0% of the patients being women.

The table also shows that 46.2% of the patients had previously been infected with COVID-19 before taking the vaccine. Of the patients who received the vaccine, 19.3% were infected with COVID-19 after taking the first dose, 29.2% after taking the second dose, and 16.0% after taking the third dose. Regarding the complaints reported by the patients, 54.2% reported fever, while 63.9% reported malaise. Headache was reported by 55.7% of the patients, and cough was reported by 25.8% of the patients. Only 10.5% of patients reported pneumonia, while 11.3% of patients reported no complaints. Additionally, 52.1% of patients were able to identify the source of their COVID-19 infection, and 84.5% had already recovered from their infection. Out of the total sample, 38.0% of the patients received treatment for their symptoms.

Table 2 provides information about the characteristics of olfactory and gustatory dysfunctions in the study population. The majority (54.8%) of participants reported chemosensory dysfunction (CD), while olfactory dysfunction was reported by 286 participants. Among those, 29.8% had anosmia, 16.8% had hyposmia, and 8.0% had parosmia, while 45.4% reported no change in olfactory function. The duration of olfactory dysfunction varied, with 45.1% of participants reporting symptoms for 1–7 days and 11.9% reporting symptoms for 61 days or more. The mean degree of olfactory dysfunction was 4.76 ± 3.16 (95% CI = 4.4–5.1), indicating a moderate-to-severe level of dysfunction.

The majority of participants (59.9%) reported changes in olfactory function after diagnosis, while 40.1% reported changes before diagnosis. Among those who reported symptoms (n = 186), fever (64.0%) and headache (70.4%) were the most reported, while pneumonia (10.8%) was the least common. After the changes in olfactory function were observed, 60.6% of participants reported improvement, while 33.1% reported worsening of symptoms. At the time of the study, olfactory dysfunction had resolved in 88.2% of participants, while 11.8% reported that the condition had not resolved.

The results presented in Table 3 indicate a significant association between COVID-19 vaccination status and olfactory dysfunction. Among the fully vaccinated individuals (n = 315), 68.6% did not report olfactory dysfunction, while 31.4% reported experiencing olfactory dysfunction.

For the partially vaccinated group (n = 107), 83.2% did not report olfactory dysfunction, while 16.8% reported olfactory dysfunction. Among the unvaccinated individuals (n = 102), 72.5% did not report olfactory dysfunction, while 27.5% reported olfactory dysfunction.

The chi-square test was conducted to assess the relationship between COVID-19 vaccination status and olfactory dysfunction. The chi-square value of 8.5166 with a *p*-value of 0.014 indicates a statistically significant association. These findings suggest that being fully or partially vaccinated may provide some level of protection against olfactory dysfunction compared to being unvaccinated. It is important to note that the majority of individuals in all groups did not experience olfactory dysfunction.

Table 4 presents the awareness and adherence of the study participants regarding olfactory exercise. The table shows that 39.1% of the participants were aware of olfactory training, while 60.9% were not aware. Among the participants who performed olfactory training, 68.9% reported an improvement in their sense of smell, while 24.2% reported no changem and 5.3% reported worsening. In terms of adherence to the olfactory exercise protocol, 32.0% of the participants reported high adherence, 45.6% reported moderate adherence, 18.4% reported low adherence, and 4.0% reported no adherence. These findings suggest that awareness and adherence to olfactory training vary among study participants, and that adherence to the exercise protocol may be associated with improvement in sense of smell.

Table 5 presents the comparison of improvement in the sense of smell between participants who adhered to the protocol and those who did not, along with the chi-square value and *p*-value. The table shows that out of the 240 participants who adhered to the protocol, 165 (68.8%) reported improvement in their sense of smell, while only 3 (30.0%) out of the 10 non-adherent participants reported improvement. The chi-square value of 8.2308 with a *p*-value of less than 0.016 indicates a statistically significant association between adherence to the protocol and improvement in the sense of smell. Therefore, it can be concluded that participants who adhered to the olfactory exercise protocol were more likely to report improvement in their sense of smell compared to those who did not adhere to the protocol.

Table 6 presents the logistic regression analysis of the association between adherence to olfactory training and duration of olfactory dysfunction, with a sample size of 286 participants. The table includes the beta coefficient, standard error, z-value, and *p*-value for each variable, as well as the odds ratio (OR) and 95% confidence interval (CI). The results show that adherence to olfactory training has a significant positive association with improvement in olfactory dysfunction (beta = 0.85, *p* < 0.001, OR = 2.34, 95% CI = 1.36–4.02). On the other hand, the duration of olfactory dysfunction has a significant negative association with improvement in olfactory dysfunction (beta = −0.27, *p* = 0.002, OR = 0.76, 95% CI = 0.62–0.93). The constant in the model is also significant (beta = −2.03, *p* < 0.001, OR = 0.13), indicating that other variables not included in the model may also be associated with improvement in olfactory dysfunction.

## 4. Discussion

The current study investigated the effectiveness of Olfactory training in improving the sense of smell in individuals who experienced a loss of smell due to COVID-19. The results suggest that olfactory training may be a viable intervention for individuals with COVID-19-related anosmia. This finding aligns with previous research that has demonstrated the positive effects of olfactory training on olfactory function in various populations, including those with post-viral olfactory dysfunction (OD).

A study conducted by researchers from the University of Dresden supported the efficacy of olfactory training in improving olfactory function in individuals with COVID-19-related anosmia [31,40]. The study involved 20 patients who were randomly assigned to either an olfactory training group or a control group. The olfactory training group performed daily training sessions, which included sniffing four different odorants twice a day for 12 weeks, while the control group did not engage in any training. At the end of the study, the olfactory training group exhibited significant improvements in olfactory function compared to the control group, which resemble the results of the current study.

It is noteworthy that the COVID-19 vaccine has been associated with a reduced occurrence of olfactory impairment in individuals who contract the virus. Several studies have indicated that vaccinated individuals who do experience COVID-19 are less likely to suffer from loss of smell or other olfactory impairments compared to those who are unvaccinated [41]. This suggests a potential protective effect of the vaccine on the olfactory system, which may mitigate the impact of the virus on the sense of smell. However, further research is needed to fully understand the relationship between COVID-19 vaccination and olfactory impairment, including long-term effects and underlying mechanisms. It is important to emphasize that the COVID-19 vaccine provides numerous other benefits, such as reducing illness severity and preventing hospitalization, in addition to potentially protecting against olfactory impairment [42,43].

Studies focusing on post-viral olfactory dysfunction (PVOD) have consistently reported the efficacy of olfactory training in comparison to spontaneous recovery. PVOD is a common symptom of viral infections, including COVID-19, and it significantly affects the quality of life of affected individuals. Damage to the olfactory system can also have psychological implications, as it is closely connected to the limbic system responsible for emotions and memory formation [44,45,46].

A randomized controlled trial examining the effectiveness of olfactory training compared to supportive therapy in patients with post-infectious OD demonstrated that olfactory training led to significant improvements in olfactory function compared to supportive therapy [47]. In addition to enhancing olfactory function, olfactory training has shown positive effects on the psychological well-being of individuals with post-viral OD [48]. This improvement in psychological well-being may be attributed to the restoration of olfactory function, which is closely linked to emotional processing and memory formation [49].

Olfactory training has also been found to promote the regeneration of olfactory receptor neurons by stimulating the proliferation of basal cells [50]. In a study conducted on mice, olfactory training was shown to increase the proliferation of basal cells and the regeneration of olfactory receptor neurons following injury to the olfactory epithelium [51]. Furthermore, olfactory training has been found to enhance synaptic plasticity in the brain [52]. Synaptic plasticity is the brain’s ability to change the strength and efficacy of synapses in response to activity, and it is believed to underlie learning and memory processes [53]. It is possible that olfactory training enhances synaptic plasticity in the olfactory bulb, thereby improving olfactory function by facilitating the processing of olfactory information in the brain [54,55].

Adherence to olfactory training has been identified as a critical factor in achieving successful treatment outcomes for post-viral olfactory dysfunction (PVOD) [56]. A study by Tara et al. (2022) demonstrated that patients who consistently adhered to olfactory training sessions (completing more than 60% of sessions) had a higher likelihood of improvement in olfactory function compared to those with lower adherence rates [57]. Consistent adherence to olfactory training is likely necessary for the regenerative processes, such as the regeneration of olfactory receptor neurons, to occur and for patients to fully recover their olfactory function [58].

Determining the optimal duration of olfactory training therapy remains a subject of debate, particularly in the context of COVID-19-related olfactory dysfunction (OD) [59]. Future studies are needed to explore the role of ongoing inflammation in post-COVID-19 OD and its implications for olfactory training outcomes. Delaying the onset of olfactory training after the onset of OD may decrease the likelihood of a successful outcome. Investigating the potential interaction between ongoing inflammation and the timing of olfactory training initiation may help identify an optimal time frame for positive outcomes. Future studies could aim to identify a “sweet spot” that maximizes the likelihood of successful outcomes by examining the interplay between these two independent mechanisms.

## 5. Limitation

Despite the concern about self-reporting and adherence to olfactory training, it is important to recognize that our study focused on the association between olfactory dysfunction and the potential benefits of olfactory training, rather than solely relying on participants’ ability to perform the training correctly or consistently. The primary objective was to investigate olfactory dysfunction following COVID-19 and explore the potential benefits of olfactory training, rather than evaluating participants’ compliance with the training. By capturing self-reported data on the presence of olfactory dysfunction, changes in symptoms, and participants’ awareness of olfactory training, we gained valuable insights into the impact of this training on the sense of smell. Additionally, the observational study design was not capable of providing high-level evidence of causality.

## 6. Conclusions

The current exploratory study showed that training on smell improves the return to the state before infection with COVID-19 disease, and it may be affected by adherence to the training as well as the duration of the exercise. Commitment and duration affect each other, which leads to a speedy recovery of the sense of smell.

## Figures and Tables

**Table 1:** Baseline characteristics of the patients (n = 524).

		Frequency	Percent
Age (Years)	18–25	211	40.3
	26–35	105	20.0
	36–45	122	23.3
	46 and more	86	16.4
Gender	Female	346	66.0
	Male	178	34.0
Infected with COVID-19 before taking vaccine		242	46.2
Infected with COVID-19 after taking first dose		101	19.3
Infected with COVID-19 after taking second dose		153	29.2
Infected with COVID-19 after taking third dose		84	16.0
Complaints reported by the patients	Fever	284	54.2
	Chills	184	35.1
	Malaise	335	63.9
	Cough	135	25.8
	Headache	292	55.7
	Nasal congestion	78	14.9
	Rhinorrhea	100	19.1
	Gastrointestinal distress	56	10.7
	Pneumonia	55	10.5
	Other	12	19.8
	None	59	11.3
COVID-19 infection source identifiable		273	52.1
Current COVID-19 infection status	Active	81	15.5
	Recovered	443	84.5
Patients received treatment		199	38.0

**Table 2:** Olfactory and gustatory dysfunctions characteristics.

		N	%
Chemosensory dysfunction (CD) reported		287	54.8
Olfactory dysfunction (n = 286)	Anosmia	156	29.8
	Hyposmia	88	16.8
	Parosmia	42	8.0
	No change	238	45.4
Duration of olfactory dysfunction (n = 286)	1–7 days	129	45.1
	8–14 days	67	23.4
	15–21 days	17	5.9
	22–29 days	16	5.6
	30–37 days	10	3.5
	38–45 days	7	2.4
	46–52 days	2	0.7
	53–60 days	4	1.4
	61 days and more	34	11.9
The degree of olfactory dysfunction	Mean: 4.76 ± 3.16 (95% CI = 4.4–5.1)		
Time of Olfactory changes observed	After diagnosis	172	59.9
	Before diagnosis	115	40.1
Reported symptoms (n = 186)	Fever	119	64.0
	Chills	62	33.3
	Malaise	83	39.0
	Cough	101	54.3
	Headache	131	70.4
	Nasal congestion	82	44.1
	Rhinorrhea	75	40.3
	Gastrointestinal distress	49	26.3
	Pneumonia	20	10.8
	Other	7	3.8
Condition of the patients after the Olfactory changes was observed	No change	18	6.3
	Worsened	95	33.1
	Improved	174	60.6
Status of olfactory dysfunction	Not resolved	34	11.8
	Resolved	253	88.2

**Table 3:** Association Between COVID-19 Vaccination Status and Olfactory Dysfunction.

	No (n = 238)	Yes (n = 286)	Total (n = 524)	Chi-Square	*p*
Fully Vaccinated (n = 315)	216 (68.6%)	99 (31.4%)	315 (60.1%)	8.5166	0.014
Partially Vaccinated (n = 107)	89 (83.2%)	18 (16.8%)	107 (20.4%)		
Unvaccinated (n = 102)	74 (72.5%)	28 (27.5%)	102 (19.5%)		
Total (n = 524)	379 (72.3%)	145 (27.7%)	524		

**Table 4:** Awareness and adherence of the study participants regarding olfactory exercise.

		N	%
Awareness of Olfactory training Responses Percentage	Yes	205	39.1%
	No	319	60.9%
Improvement in Sense of Smell Responses Percentage	Improved	168	68.9%
	No Change	59	24.2%
	Worsened	13	5.3%
Adherence to Olfactory Exercise Protocol Responses Percentage	High Adherence	80	32.0%
	Moderate Adherence	114	45.6%
	Low Adherence	46	18.4%
	No Adherence	10	4.0%

**Table 5:** Comparison of Improvement in Sense of Smell Between Participants Who Adhered to the Protocol and Those Who Did Not.

Adherence to Protocol	Improved	No Change	Worsened	Total
Yes	165	55	20	240
No	3	4	3	10
Total	168	59	23	250
Chi-Square value	8.2308			
*p*-value	0.016			

**Table 6:** Logistic Regression Analysis of Adherence to Olfactory training and Duration of Olfactory Dysfunction (n = 286).

Variable	Beta	SE	Z Value	*p*-Value	OR	95% CI
Adherence to Olfactory training	0.85	0.27	3.15	0.002	2.34	1.36–4.02
Duration of Olfactory Dysfunction	−0.27	0.09	−3.10	0.002	0.76	0.62–0.93
Constant	−2.03	0.52	−3.93	<0.001	0.13	-

## Data Availability

Data available upon request.
